# Mechanism of Werner DNA Helicase: POT1 and RPA Stimulates WRN to Unwind beyond Gaps in the Translocating Strand

**DOI:** 10.1371/journal.pone.0004673

**Published:** 2009-03-05

**Authors:** Byungchan Ahn, Jae Wan Lee, Hana Jung, Gad Beck, Vilhelm A. Bohr

**Affiliations:** 1 Department of Life Sciences, University of Ulsan, Ulsan, Korea; 2 Laboratory of Molecular Gerontology, Biomedical Research Center, National Institute on Aging, National Institutes of Health, Baltimore, Maryland, United States of America; University of Florida, United States of America

## Abstract

WRN belongs to the RecQ family of DNA helicases and it plays a role in recombination, replication, telomere maintenance and long-patch base excision repair. Here, we demonstrate that WRN efficiently unwinds DNA substrates containing a 1-nucleotide gap in the translocating DNA strand, but when the gap size is increased to 3-nucleotides unwinding activity significantly declines. In contrast, *E. coli* UvrD (3′→5′ helicase), which recognizes nicks in DNA to initiate unwinding, does not unwind past a 1-nucleotide gap. This unique ability of WRN to bypass gaps supports its involvement in DNA replication and LP-BER where such gaps can be produced by glycosylases and the apurinic/apyrimidinic endonuclease 1 (APE1). Furthermore, we tested telomere repeat binding factor 2 (TRF2), both variants 1 and 2 of protector of telomeres 1 (POT1v1 and POT1v2) and RPA on telomeric DNA substrates containing much bigger gaps than 3-nucleotides in order to determine whether unwinding could be facilitated through WRN-protein interaction. Interestingly, POT1v1 and RPA are capable of stimulating WRN helicase on gapped DNA and 5′-overhang substrates, respectively.

## Introduction

Human RecQ helicases maintain genomic integrity through their involvement in diverse aspects of DNA metabolism including DNA replication, DNA repair, recombination, and telomere maintenance. The genomic instabilities associated with cells deficient in *RECQ2*, *RECQ3*, *and RECQ4* genes are linked to the rare genetic disorders, Bloom syndrome, Werner syndrome (WS), and Rothmund-Thompson syndrome, respectively. WS causes an early onset of aging in patients following puberty with physical features that are strikingly similar to normal aging. WS patients exhibit graying and loss of hair, bilateral cataracts, atherosclerosis, diabetes mellitus type II, osteoporosis, hypogonadism, and a propensity to develop sarcomas [1]. In addition, WS cells exhibit genomic instability [2], replication defects [3], aberrant telomere maintenance [4], and a gene expression profile that resembles normal human aging [5], implicating the role of Werner syndrome protein (WRN) in maintenance of self-renewing capabilities and prevention of cellular senescence. In most cases of WS, frameshift mutations resulting in truncated WRN proteins are the leading cause [6]–[8].

Cells from WS patients grow slowly and senesce prematurely in culture. This can be reversed by expression of the catalytic telomerase subunit of human telomerase reverse transcriptase (hTERT), demonstrating a critical role of WRN in the process of cellular senescence by maintaining the integrity of telomeres [9]. Furthermore, primary fibroblasts from WS patients exhibit loss of lagging strand telomeres which can be reconstituted with a nuclease-deficient WRN-E84A but not with the helicase-deficient WRN-K577M. This associates WRN helicase function with sustaining lagging strand telomeres [4]. Therefore, it is not surprising that WRN physically interacts with proteins associated with telomeres. Telomere binding protein 2 (TRF2) stimulates WRN and BLM helicase activities [10]. Likewise, the telomeric single-strand DNA-binding protein, POT1 (both variants POT1v1 and POT1v2), stimulates WRN unwinding of telomeric forks and synthetic D-loop substrates [11]. Another protein, replication protein A (RPA), stimulates WRN helicase activity dramatically and this functional stimulation is mediated by the direct acidic repeat and the RecQ C-terminal (RQC) domains of WRN [12], [13].

WRN helicase translocates along DNA strands in the 3′→5′ direction [14]. A number of studies have shown that WRN can unwind a wide variety of different oligonucleotide-based DNA substrates, including forked duplexes, four-way junctions modeling the Holliday junction, and simple 3′-tailed duplexes in addition to non-B-form DNA structures, such as G-quadruplexes [15]. Furthermore, WRN is a unique protein in the RecQ helicase family because it also possesses 3′→5′-exonuclease activity [16]. WRN has preferential DNA binding activities toward DNA substrates that possess both single-stranded DNA (ssDNA) and double-stranded DNA (dsDNA) junctions [17]. In addition, the WRN conserved RQC (RecQ C-terminal) domain has been shown to be the strongest DNA binding region with position K1016 mediating important WRN-DNA interaction which inactivates WRN helicase function when mutated [17], [18]. The preference of WRN for these particular DNA substrates directly translate to preferential DNA unwinding activity of WRN toward structure-specific DNA substrates such as bubbles, forks, Holliday junctions, G4 tetraplexes, triple helices, blunt ended or 5′-overhang linear DNA substrate.

Previous studies on the biochemical characterization of WRN helicase suggest that WRN requires at least 8-nucleotide (nt) 3′-ssDNA tail to properly load and initiate unwinding [19]. In addition, WRN must interact with at least a 3-nt 5′-ssDNA tail at the fork junction [19]. Also, bulky adducts such as benzo[a]pyrene 7,8-diol 9,10-epoxide, a cellular metabolite of benzo[a]pyrene produced by UDP-glucuronosyltransferase mediated glucuronidation, inhibits WRN helicase activity only when adducts are present in the translocating DNA strand of WRN [20]. However, the DNA unwinding properties of WRN are poorly understood when in the absence of a 3′-ssDNA tail or in the presence of gaps in the translocating strand. WRN may encounter such gaps frequently *in vivo* because gaps arise during intermediary steps of DNA repair processes. For instance, such gaps are found after dual incisions mediated by ERCC1-XPF during nucleotide excision repair and interstrand cross-link repair, also during mismatch repair downstream of EXO1 catalyzed removal of nucleotides, and subsequent steps following APE1-catalyzed DNA backbone cleavage in BER [21]–[24]. Thus, we hypothesized that gaps present in the translocating DNA strand would pose an obstacle for WRN helicase progression. Furthermore, WRN interacts physically and functionally with many DNA binding proteins involved in DNA repair processes. If present at DNA gaps, these proteins would bind to the exposed ssDNA or dsDNA in the vicinity of the gap and could modulate the catalytic function of WRN. For example, single strand binding proteins such as replication protein A (RPA) coat the 5′-overhang and recruit interacting proteins, among which WRN could be recruited, thereby modulating the WRN helicase activity. In addition, DNA damages in human telomeres consisting of 5–15 kb of TTAGGG tandem repeats could give rise to gaps. The telomeric DNA is highly susceptible to DNA damage from oxidative stress or alkylating agents suggesting that DNA repair processes occur in order to maintain genomic stability in telomeric DNA [25]. Since WRN functionally interacts with telomeric DNA binding proteins such as telomere repeat binding factor 2 (TRF2), or protector of telomeres 1 (POT1), we investigated whether they could facilitate WRN unwinding across the gap by acting as a bridge or a recruitment factor in the absence of a 3′-ssDNA tail for loading.

## Methods

### Recombinant proteins

Recombinant His_6_-tagged WRN protein (WRN and WRN-E84A) was over-expressed in SF*9* insect cells after transfection with WRN-E84A baculovirus or WRN baculovirus. WRN and WRN-E84A protein was purified as previously described [26]. WRN-HR was cloned into a Gateway BaculoDirect Baculovirus Expression System (Invitrogen) according to the manufacturer's guidelines and the recombinant N-terminal His_6_-tagged proteins were purified using Nickel-desalting-gel filtration columns attached to AKTA Express FPLC (Amersham). Recombinant N-terminal His_6_-tagged TRF2 was prepared as described previously [10]. Recombinant POT1v2 was a gift from Dr. Thomas R. Cech (University of Colorado). Recombinant human RPA was provided by Dr. Mark Kenny (Albert Einstein Cancer Center, New York). Recombinant *E. coli* UvrD was purchased from BioHelix and stored in 20 mM Tris pH 8.3, 200 mM NaCl, 50% glycerol, 1 mM disodium EDTA, 25 mM 2-ME.

### DNA substrate preparation

The 34 bp duplex DNA fork substrate with 3′ and 5′-single stranded T_15_ overhangs were prepared as described previously [27]. The gap substrates were prepared by annealing 5′-^32^P-radiolabeled strand oligomer (as indicated in the figures) to two other strand oligomers by adding 3 to 4- fold excess unlabeled strand oligomer. Table 1 shows sequences of top and two bottom oligomers that were used to generate a specific DNA substrate containing a gap. For the annealing reaction, radiolabeled oligomer was incubated with other two oligomers at 95°C for 5 min. The reaction mixture was allowed to cool down to room temperature over next 3 h.

**Table 1:** Sequences of oligomers used for the making of DNA substrates.

1nt-Gap	Top	5′-TTTTTTTTTTTTTTTTTCGATTACGCGTTACGCGTTACGCGCATG CACTAC-3′
	Bottom1	5′-CGTAACGCGTAATCGTTTTTTTTTTTTTTT-3′
	Bottom2	5′-GTAGTGCATGCGCGTAAC-3′
2nt-Gap	Top	Same as for 1ntGap Top
	Bottom1	Same as for 1ntGap Bottom1
	Bottom2	5′-GTAGTGCATGCGCGTAA-3′
3nt-Gap	Top	Same as for 1ntGap Top
	Bottom1	Same as for 1ntGap Bottom1
	Bottom2	5′-GTAGTGCATGCGCGTA-3′
No-gap	Top	5′-TTTTTTTTTTTTTTTTTCGATTACGCGTTACGCGTTACGCGCATG CACTAC-3′
	Bottom	5′- GTAGTGCATGCGCGTAACG CGTAACGCGTAATCGTTTTTTTTTTTTTTT-3′
TRF2 substrate	Top	5′-TTTTTTTTTTCCTTATTACGCGTTACGCGTTTAGGGTTAGGG TTAGGGTTAGGG-3′
	Bottom1	5′-TTTTTTTTTTGGAATAATGCGCAATGC-3′
	Bottom2	5′-AATCCCAATCCCAATCCCAATCCC-3′
POT1 substrate	Top	5′-TTTTTTTTTTCCTTATTACGCGTTATTAGGGTTAGGGTTAGG GTTAGGGTTAGGGCGCGCATGCACTACGG-3′
	Bottom1	5′-TTTTTTTTTTGGAATAATGCGCAAT-3′
	Bottom2	5′-GCGCGTACGTGATGCC-3′

### Helicase assay

The reaction conditions for helicase assays were performed as described previously [28]. 10 µl reactions contained 30 mM HEPES (pH 7.4), 5% glycerol, 40 mM KCl, 100 ng/µl BSA, 0.8 nM DNA substrate, 2 mM MgCl_2_ and 2 mM ATP. Reactions were incubated for 15 min at 37°C and terminated using 5 µl stop buffer (0.05 M EDTA, 40% glycerol, 1% SDS, 0.05% bromophenol blue, and 0.05% xylene cyanol FF). A cold trap oligomer (100-fold) complementary to the unlabeled, unwound oligomer was added at the end of the enzymatic reaction. Reaction mixtures were electrophoresed in non-denaturing 12% polyacrylamide gels in 1× TAE or TBE buffer and the results were analyzed using Molecular Dynamics phosphorimager (ImageQuant software) or Scion image.

## Results

### The WRN helicase efficiently unwinds past 2 nucleotide gaps in the translocating DNA strand

The WRN helicase possesses a unique property for DNA binding to junctions containing both ssDNA and dsDNAs [17]. It requires at least 3 nt 5′-ssDNA and 8 nt 3′-ssDNA tails at the fork junction for optimal helicase activity [19]. Previously, WRN has been shown to unwind 1-nt gapped substrate with moderate efficiency even in the presence of 3′-internal mismatches [29]. However, this interesting observation was not characterized further. In order to investigate WRN gap-unwinding activity mechanistically, we designed forked substrates with increasing gap-size in the translocating DNA strand.

**Figure 1:**
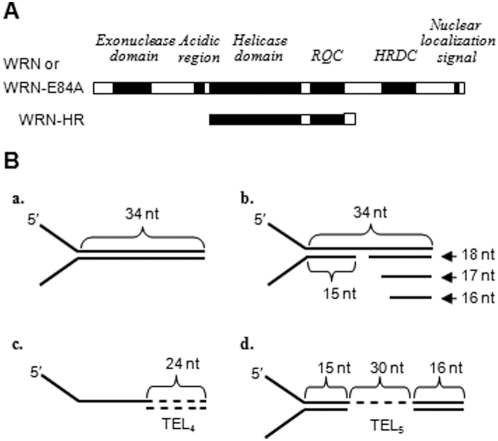
A schematic diagram of recombinant proteins and DNA substrates. (A) The modulations of active helicases were studied using WRN variants. WRN-E84A is a full-length WRN with the N-terminal exonuclease inactivating mutation at E84 to alanine. WRN-HR is a WRN fragment containing helicase and RQC domains. (B) All DNA substrates contained 5′-10-nt poly-T tails while 3′-10-nt poly-T tails were excluded only in few of the indicated experiments. [a] Fork substrate without gap. [b] Fork substrate with 1 to 3-nt gap. [c] Fork substrate with 4 telomeric repeats located in the duplex DNA following 3-nt gap. [d] Fork substrate with 5 telomeric repeats located in ssDNA of the non-translocating strand.

**Figure 2:**
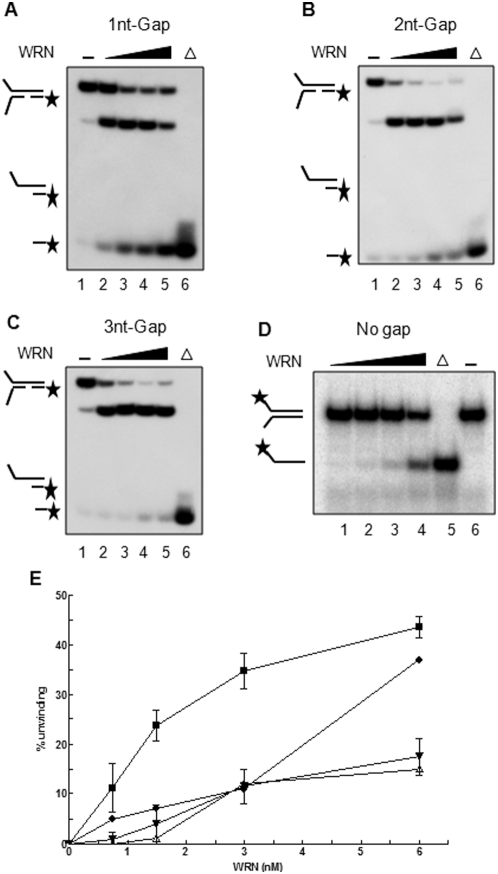
Radiometric DNA helicase assay using gap substrates and WRN. Each assay was performed using 1.5 nM of each substrate and a range of WRN concentration from 0.75 to 6 nM. A cold trap oligomer complementary to the unlabeled, unwound oligomer (100-fold) was added at the end of the enzymatic reaction. (A) A representative gel picture of the assay using 1-nt gap. The arrows indicate the migration pattern of the unwound product (radiolabeled ss oligomer). (B) A representative gel picture of the assay using 2-nt gap substrate. (C) A representative gel picture of the assay using 3-nt gap substrate. (D) A representative gel picture of the assay using DNA substrate without a gap. (E) Quantitations of at least three or two independent experiments shown in A and B. The graph is plotted as the percentage of unwound product on the Y-axis and the concentration of WRN on the X-axis. The mean values of three different experiments were plotted with standard deviation indicated by *error bars* (1-nt gap substrate and 2-nt gap substrate). Filled-circles indicate DNA substrate without a gap. Likewise, filled-squares indicate 1-nt gap substrate, filled-inverse triangles indicate 2-nt gap substrate and open triangles, 3-nt gap substrate. Asterisk indicates a 5′-^32^P labeled strand. Δ indicates boiled substrate.

We used a long-forked substrate as a control for WRN helicase activity as described previously and modified the substrate generating gaps of different sizes on the translocating strand. The gaps are 1-, 2-, and 3-nt, located 15 bp from the fork, and consequently resulting in different size downstream duplex (Fig. 1B-b). We then performed helicase assays using recombinant WRN proteins and the DNA substrates. Figures 2A–2D show representative helicase assays with WRN and gap DNA substrates and substrates without gaps. The unwinding of gapped substrates increased in a WRN concentration-dependent manner as shown in Figure 2A–2D. At a 6 nM concentration of the WRN protein, quantifications of these assays revealed that WRN could unwind ∼40% of the 1-nt gap substrate (the mean of three different experiments), ∼17% of the 2-nt gap substrate (the mean of three different experiments), and ∼15% of the 3-nt gap substrate (the average of two different experiments) (Fig. 2E). In the absence of ATP, no unwinding of the 1-nt gap substrate was observed (data not shown). The less efficient unwinding of the 2-nt gap or 3-nt gap compared to no gap suggested that WRN could not tolerate a gap up to 2-nt in the translocating DNA strand. Interestingly, much higher unwinding efficiency of WRN on the 1-nt gap substrate than on the substrate with no gaps suggested that WRN helicase is able to recognize and translocate over a 1-nt gap.

**Figure 3:**
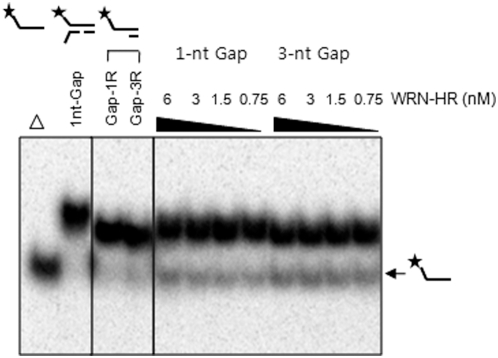
Radiometric DNA helicase assay using gap substrates and WRN-HR. Lanes 1–4 are control samples without WRN-HR. Lanes 5–8 contain decreasing concentration of WRN-HR from 6 nM to 0.75 nM using 1-nt gap substrate. Likewise, lanes 9–12 contain the same range of WRN-HR concentration using 3-nt gap substrate. Asterisk indicates a 5′-^32^P labeled strand. Δ indicates boiled substrate.

**Figure 4:**
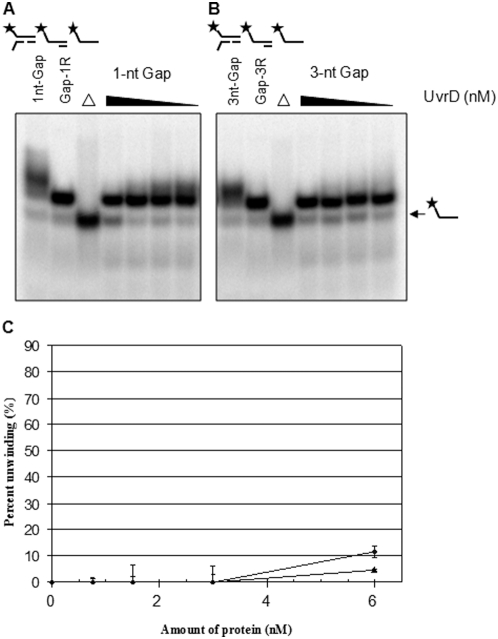
Radiometric DNA helicase assay using gap substrates and UvrD. The equal molar concentration range of UvrD was used from 0.75, 1.5, 3 to 6 nM. The arrows indicate the migration pattern of the unwound product (radiolabeled ss oligomer). (A) A representative gel picture of the assay using 1-nt gap. (B) A representative gel picture of the assay using 3-nt gap substrate. (C) Quantitations of at least two or more independent experiments shown in A and B. The graph is plotted as the percentage of unwound product on the Y-axis and the concentration of WRN on the X-axis. The mean values of replicate experiments were plotted with standard deviation indicated by *error bars*. Likewise, filled-diamond indicate 1-nt gap substrate, filled-triangle indicate 3-nt gap substrate. Asterisk indicates a 5′-^32^P labeled strand. Δ indicates boiled substrate.

To determine which regions of WRN are responsible for the unwinding of gapped substrates, we performed helicase assays using the WRN-HR protein fragment that spans from amino acid (aa) 500 to 1092 of WRN, containing both helicase and RQC domains (termed as HR, Fig. 1A). Previous results demonstrated that the unwinding activity of WRN-HR was similar to that of the full-length WRN using DNA substrates that are shorter than 50-nt long without gaps [30]. Thus, we hypothesized that WRN-HR also would be as efficient in unwinding 1-nt gap substrates as the full-length WRN. However, we observed no unwinding of the 1- to 3-nt gap substrates with WRN-HR, suggesting that other DNA binding domains apparently play critical roles in unwinding gapped substrates (Fig. 3). Radiometric helicase assays were then performed using *E. coli* DNA helicase, UvrD, which has been implicated in mismatch and nucleotide excision repair (NER) pathways [31], [32]. UvrD can initiate DNA unwinding at nicks in DNA without requiring other accessory proteins [33], suggesting that it has the potential to unwind gapped substrates. However, UvrD was very poor at unwinding 1- to 3-nt gapped substrates as shown in Figure 4A and 4B. Quantifications of the unwound product from at least two independent experiments showed less than 12% unwinding of 1-nt gap substrate compared to ∼50% unwinding by WRN (comparison of Fig. 4C to Fig. 2E). This suggests that WRN possesses a unique ability to efficiently translocate over a 1-nt gap in the translocating strand.

### RPA can recruit WRN to DNA and stimulate it to initiate unwinding in the absence of a 3′-ssDNA tail normally required for loading

**Figure 5:**
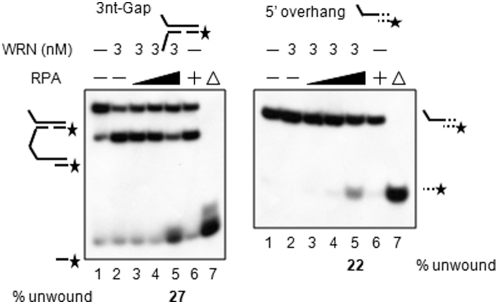
Radiometric DNA helicase assay using gap substrates with WRN in the presence of RPA. (A) Lanes 3–5 contained WRN (3 nM) and a range of RPA (6 nM, 12 nM, and 24 nM) concentrations with 3-nt gap substrates. Lane 2 contained only WRN (3 nM) and lane 6 contained only RPA (24 nM). Lane 1 contained control DNA substrates without any proteins and the substrates were boiled (lane7). (B) The reaction mixtures contained DNA substrate as indicated in the upper right hand corner of the figure which is exactly the same substrate shown in Figure 1B[c] except for lacking the 3′-ss tail oligo. Lane 1 was a control reaction with only DNA substrate. Lane 7 was a heat-denatured substrate. Lane 2 was a control sample with only WRN. Lane 6 was a control samples with only RPA in the concentration range of 24 nM. Lanes 3–5 were reaction mixtures containing both RPA and WRN. WRN concentration was held constant at 3 nM and the RPA concentration was in the range of 6 nM to 24 nM. At the end of the enzymatic reaction, a cold-trap” oligomer complementary to the unlabeled, unwound oligomer was added. The arrows indicate the migration pattern of the unwound product (radiolabeled top strand oligomers). Asterisk indicates a 5′-^32^P labeled strand. Δ indicates boiled substrate.

WRN exhibits progressive decrease in its ability to unwind gapped substrates with increasing gap size. Thus, we hypothesized that a prominent WRN-interacting protein may act as an accessory protein to facilitate unwinding over a gap bigger than 3-nt in length. Such longer gaps might arise during LP-BER, and replication protein A (RPA) is a known processivity factor for WRN helicase involved in this process. A helicase assay on 3-nt gap substrates was carried out in the presence of WRN and RPA. As shown in Figure 5A, there was a significant stimulation of unwinding past a 3-nt gap in the presence of RPA (compare lane 2 to lane 5). This suggested that WRN helicase could be facilitated to translocate over the 3-nt gap by the WRN-RPA interaction. In order to clarify whether RPA is capable of directly recruiting WRN without the minimum length of a 5-nt 3′-ss tail required by WRN alone [19] to load onto the DNA substrate, we designed a different DNA substrate using four telomeric repeats as shown in Fig. 1B-c except that the substrate lacked a 3′-ss tail (lacking bottom 1 oligomer in TRF2 substrate, Table 1). The result of a helicase assay using this substrate showed a clear unwinding of the duplex DNA region in the presence of RPA (Fig. 5B, lane 5 compared to lanes 2 and 6), but its extent is slight. WRN unwinding was 22% for wild-type WRN at the highest RPA concentration used in lane 5 (Fig 5B).

### POT1v1 can help WRN unwind past the gap through protein-protein interaction

Recently, WRN was determined to be a functional partner of protection of telomeres 1 (POT1) protein, which is a part of the shelterin complex [11], [34]. POT1 appears to play an integral role in capping the single-stranded telomere ends [35]. POT1v2 is a C-terminal truncated form of POT1v1, and both v1 and v2 have been shown to bind efficiently to single-stranded telomeric DNA through the N-terminal oligonucleotide/oligosaccharide binding (OB) folds [35]. The minimum length requirement for the DNA binding of POT1 has been determined to be a 10-nt with a sequence of “TTAGGGTTAG” from the X-ray co-crystal structure of POT1v2 and ssDNA [11]. Since POT1 binds to single-stranded DNA, POT 1 functions somewhat like RPA, but is telomere specific, thus comparing POT1∶WRN to RPA∶WRN will interrogate whether the reaction is telomere specific.

**Figure 6:**
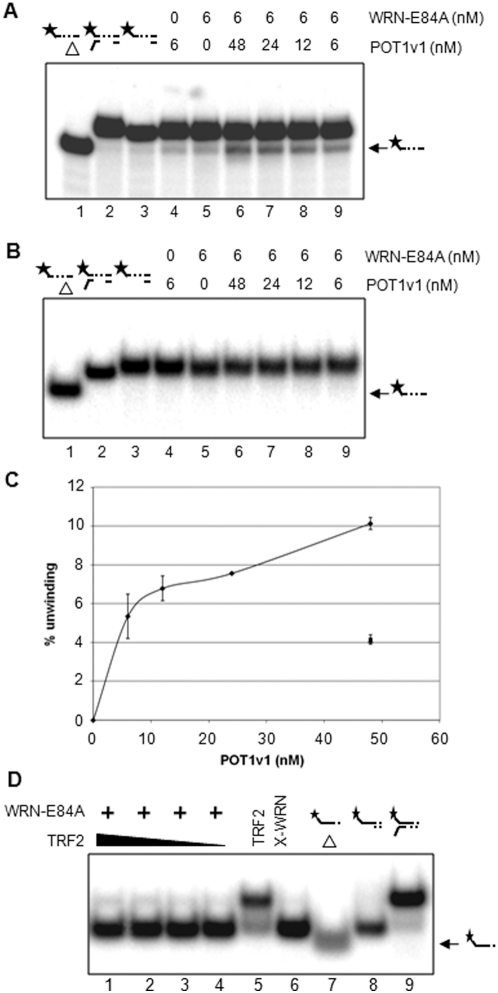
Radiometric DNA helicase assay using WRN in the presence of POT1. The concentration range of POT1 variants used in these reactions was from 6 to 48 nM. The concentration of WRN-E84A was held constant at 6 nM. The DNA substrate used for these reactions is shown in Figure 1B[d]. (A) The reaction mixtures contained POT1v1 and WRN-E84A with the DNA substrate for POT1v1 to pre-bind. (B) The reaction mixtures contained POT1v2 and WRN-E84A with the same DNA substrate as (A). (C) Quantitations of at least two or more independent experiments shown in (A) is plotted. The graph is plotted as the percent of displacement of the radiolabeled oligomer on the Y-axis and the concentration of POT1 variants on the X-axis. Filled diamond represents reaction mixtures containing POT1v1 and WRN-E84A. Filled circle represents reaction mixture containing only POT1v1 and Filled triangle only WRN-E84A. (D) The reaction mixtures contained TRF2 and WRN-E84A with DNA substrate for TRF2 to pre-bind as shown in 1B[c]. TRF2 was added in these reactions from 12 nM (lane 4), 25 nM (lane 3), 50 nM (lane 2) to 100 nM (lane 1) and the concentration of WRN-E84A was held constant at 6 nM. Asterisk indicates a 5′-^32^P labeled strand. Δ indicates boiled substrate.

Based on our results that WRN was very inefficient at unwinding past a 3-nt gap (Fig. 2C), we questioned whether POT1, which binds to 10-nt telomeric ssDNA (or longer), could help WRN unwind a bigger gap in the translocating strand. Thus, we designed the DNA substrate shown in Figure 1B-d which harbors 5 telomeric ssDNA repeat regions allowing sufficient room for POT1v1 to bind. We pre-bound POT1 on this substrate and asked whether POT1v1 could facilitate WRN in unwinding past the 30-nt gap. Figure 6A shows a representative gel picture of at least two independent radiometric helicase assays for which the reactions were initiated by adding WRN-E84A to DNA substrates pre-bound with POT1v1. Interestingly, POT1v1 exhibited mild yet distinct stimulation of WRN-E84A to unwind past the gap in a concentration-dependent manner. The quantification of unwound product as a result of POT1v1 stimulation shows that at the 1∶8 ratio of WRN-E84A to POT1v1, we observed ∼10% increase in the unwound product (Fig. 6C). Furthermore, when we used the same assay with a modified DNA substrate lacking a 3′-ss tail, we did not observe any increase in the unwound product suggesting that POT1 stimulates WRN-E84A through a mechanism different from direct recruitment of WRN by RPA (data not shown). The protein-protein interaction between WRN and POT1 was previously determined to be mediated by the N-terminal of POT1 while the C-terminal was dispensable for the WRN-POT1 interaction [11]. Thus, we also tested unwinding with POT1v2, which has similar DNA binding activity and WRN helicase-stimulating activities as POT1v1. The results from Figure 6B show no stimulation of WRN unwinding activities in the presence of POT1v2 using the same gapped substrates.

Next, we tested whether telomere repeat binding factor 2 (TRF2), another subunit of the shelterin complex [36] and a functional partner of WRN, could also stimulate WRN to unwind past the gap like RPA and POT1v1. As shown in Figure 1B, the DNA substrate for TRF2 contained 4 double-stranded telomeric repeats following the 3-nt gap (Fig. 1B-c) in order to accommodate proper pre-binding of TRF2 [10]. Figure 6D shows a representative gel picture of at least 3 independent experiments using the DNA substrate shown in Figure 1B-c. In contrast to the situation for RPA and POT1v1, TRF2 did not facilitate WRN unwinding beyond the gap even at the highest ratio of WRN to TRF2, 1∶16.7 (Fig 6D, lane 1). Furthermore, we positioned 4 telomeric repeats in front of the 3-nt gap in order to determine whether different positioning of the TRF2 DNA binding might help WRN unwinding past the gap, but TRF2 failed to aid WRN in this process (data not shown). This suggests that regardless of location of the TRF2 binding site before or after the 3-nt gap TRF2 cannot aid WRN to unwind past the gap.

## Discussion

**Figure 7:**
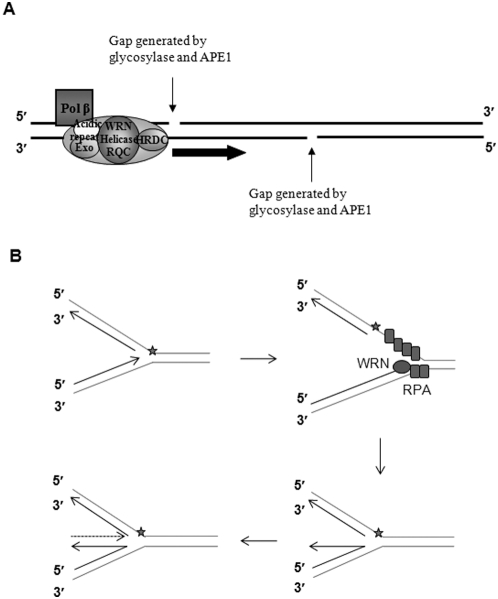
The model of unique WRN helicase mechanism in BER or LP-BER. (A) Preceding steps in the BER or LP-BER involving glycosylases and APE1 result in gaps in the translocating and non-translocating DNA strands. WRN is capable of unwinding 1- to 2-nt gaps in the translocating strand proficiently and assists the strand displacement synthesis of polymerase β during subsequent steps. (B) The fork could regress into a Holliday junction (also called a chicken foot), where the nascent leading strand serves as a template for the lagging strand synthesis.

WRN is proposed to participate in long patch base excision repair (LP-BER) based on the functional interaction with many of the proteins involved in this pathway [37], [38]. WRN is implicated in subsequent steps following processing by APE1 because while APE1 remains associated with the nicked DNA product, WRN helicase is inhibited [38]. In addition, WRN has been shown to have poor unwinding activity at a nicked DNA substrate suggesting that WRN helicase initiation at nicks in the DNA is unfavorable [19], [39]. In contrast, we here report a unique WRN helicase mechanism by which WRN catalyzes unwinding even when 1- to 2-nt gaps are present in the translocating DNA strand. The ability of WRN to catalyze gapped substrate unwinding suggests that WRN has a specific biological function connected to this DNA intermediate. Such gaps arise from the intermediary steps in the two subpathways of BER after the completion of glycosylases and APE1 catalyzed steps [38]. During replication, when the replication fork complex encounters such gaps in the leading strand, a stalled replication fork will collapse, generating a double-strand break. The ability of WRN helicase to translocate the gap would suggest another mode in which replication forks might translocate the gaps in the leading strand before collapsing. Also, taken together with previously findings, our observation of WRN unwinding of gap-sizes up to 2-nt provide support for its biological role in BER and LP-BER. WRN may be recognizing the presence of gaps and unwind over 1- to 2-nt gaps in the translocating strand while increasing the processivity of the strand displacement synthesis by DNA polymerase β (Fig. 7A). Another possibility might be that such gaps could arise from the 5′-flap incision activity of FEN-1, resulting from either the downstream LP-BER process or the removal of 5′-flap structures when Okazaki fragments are displaced in order to initiate strand displacement synthesis or to resume replication.

Previous observations that WRN requires at least a 8-nt 3′-ssDNA tail to initiate DNA unwinding argues against the idea that WRN falls off at the gap and reloads to continue unwinding downstream of the gap [19]. When *E. coli* UvrD helicase, implicated in nucleotide excision repair (NER) and mismatch DNA repair, was tested in the helicase assay using the gapped substrates, we observed almost complete lack of unwinding downstream to the gap, which is strikingly different from the situation for WRN [40]. This suggests a distinct role of WRN in unwinding past the gap and is distinguishable from the involvement with other DNA repair pathways such as NER or mismatch repair.

The mechanism by which WRN unwinds past the gaps involves the N-terminal and C-terminal parts of WRN protein based on our observations using the WRN-HR fragment. This finding is supported by the previous data obtained from our lab comparing full-length WRN protein and WRN-HR helicase activity. They diminished dramatically when the DNA substrate length was increased beyond 50-nt [30]. In support of this notion, the exonuclease and HRDC domains of WRN have been determined previously to possess auxiliary DNA binding activities [17]. Taken together, these data suggest the importance of using full-length WRN or WRN-E84A for mechanistic studies of WRN helicase function.

The WRN-RPA interaction has been studied previously revealing that the protein-protein interaction is mediated by the direct acidic repeats in addition to the RQC domain, and upon this interaction RPA acts as a processivity factor for the WRN helicase [30]. However, it has not previously been observed that RPA can act as a direct recruiting factor for WRN to initiate DNA unwinding without the required 3′-ssDNA tail.

RPA has been also implicated in the LP-BER pathway [41] and the ability of RPA to recruit WRN could be one of the mechanism by which WRN translocates to gaps in the BER intermediates in order to initiate unwinding of the DNA downstream of the lesion.

When the replication fork complex encounters a lesion in the lagging strand, DNA lesions on the lagging strand can lead to uncoupling of the replication complex and the replication complex continues to unwind as leading strand synthesis goes on. The stalling of the replication fork could be recovered by the formation of a so-called chicken foot intermediate, a structure that is analogous to a Holliday junction. RPA bound to the leading strand would recruit WRN to unwind the DNA duplex along the synthesized strand (5′→3′), allowing fork regression. Repriming replication will be then resumed (Fig. 7B).

There has been a great deal of interest in the field in the question of where in the cell WRN's main function lies. In particular, it has been proposed that WRN exerts its main function at the telomere [11], [42].). Thus, we compared the function of RPA, which works in the general genome with POT1, which works specifically at the telomere. Both of these proteins facilitated the unwinding of the 3-nt gaps. The similarity between POT1 and RPA functions with respect to WRN suggests the involvement of POT1 and WRN in the context of BER intermediary steps to resolve BER substrates present in telomeres.
